# Novel cytogenic and neurovascular niches due to blood–brain barrier compromise in the chronic pain brain

**DOI:** 10.1186/s12990-015-0066-6

**Published:** 2015-10-09

**Authors:** Maral Tajerian, J. David Clark

**Affiliations:** Veterans Affairs Palo Alto Health Care System, 3801 Miranda Ave., Palo Alto, CA 94304 USA; Department of Anesthesiology, Stanford University School of Medicine, Stanford, CA USA; Palo Alto Institute of Research and Education, Palo Alto, CA USA

**Keywords:** Blood–brain-barrier, Chronic pain, Neuroinflammation, Neurogenesis, Neovascularization, Pain-related brain plasticity, Pain-related co-morbidities

## Abstract

**Background:**

The mechanisms by which painful injuries are linked to the multitude of pain-related comorbidities and neuroplastic changes in the brain remain poorly understood. Here we propose a model that relies on epi-neuronal communication through the vascular system to effect various brain structures. Specifically, we hypothesize that the differential vulnerability of the blood–brain barrier (BBB) in different brain regions is associated with region-specific neuroplastic and neurovascular changes that are in turn associated with particular pain-related comorbidities.

**Presentation of the hypothesis:**

We will present our hypothesis by focusing on two main points: (A) chronic pain (CP) is associated with differential BBB compromise. (B) Circulating mediators leaking through the BBB create cytogenic and neovascular niches associated with pain-related co-morbidities.

**Testing the hypothesis:**

Pre-clinically, our hypothesis can be tested by observing, in parallel, BBB compromise, (neo)vascularization, neurogenesis, and their co-localization in animal pain models using imaging, microscopy, biochemical and other tools. Furthermore, the BBB can be experimentally damaged in specific brain regions, and the consequences of those lesions studied on nociception and associated comorbidities. Recently developed imaging techniques allow the analysis of blood brain barrier integrity in patients providing a route for translation of the laboratory findings. Though perhaps more limited, post-mortem examination of brains with available pain histories constitutes a second approach to addressing this hypothesis.

**Implications of the hypothesis:**

Understanding changes in BBB permeability in chronic pain conditions has clear implications both for understanding the pathogenesis of chronic pain and for the design of novel treatments to prevent chronic pain and its consequences. More broadly, this hypothesis may help us to understand how peripheral injuries impact the brain via mechanisms other than commonly studied efferent sensory pathways.

## Background

Chronic pain (CP) is associated with a multitude of comorbidities, including cognitive impairment, memory deficits, depression, and anxiety [1], which further exacerbate disability and declining quality of life in CP patients. This implies the presence of neuroplastic changes in the brain. Indeed, such evidence is available from patients [2] and preclinical models [3, 4]. What remains less clear is the mechanism by which painful injuries result in changes that are separated in time and space from the initial injury, namely delayed changes in the brain. One attractive candidate set of mechanisms are the ascending and descending neuronal pathways connecting the periphery and the brain [5], but it is unclear whether these pathways can account for the host of central changes observed in CP patients [6]. Here we propose a complimentary mechanism that relies on epi-neuronal communication through the vascular system and its effect on various brain structures.

Although the blood–brain barrier (BBB) exists throughout the arborized levels of the vascular system in the central nervous system (CNS), there is scant basis for concluding that the brain is uniformly protected from BBB compromise. We therefore hypothesize that the differential vulnerability of the BBB in different brain regions is associated with region-specific neuroplastic and neurovascular changes in the brain that parallel particular pain-related comorbidities.

## Presentation of the hypothesis

We will present our hypothesis in the following main points:

- CP is associated with differential BBB compromise.

**Fig. 1:**
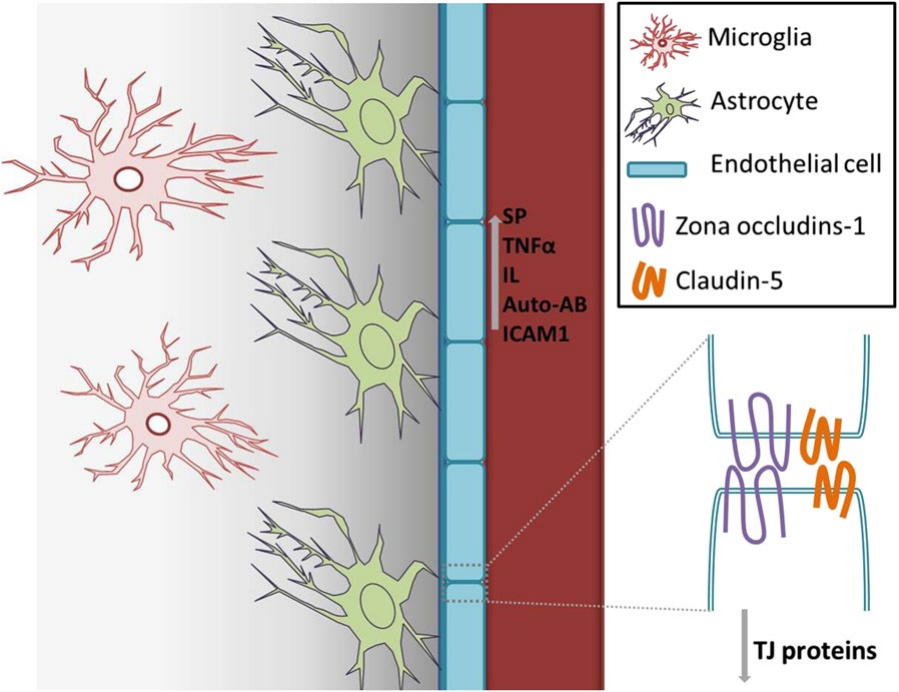
Summary of the hypothesis. *AutoAB* autoantibody, *ICAM* intercellular adhesion molecule, *IL* interleukin, *SP* substance P, *TJ* tight junction, *TNFα* tumor necrosis factor alpha

BBB integrity is altered in various painful clinical conditions [7–9] and in pre-clinical models of pain [10–13]. Most of the preclinical studies involve models of inflammatory pain, and the breakdown of tight junctions (TJ) has been implicated in the observed BBB compromise. This possibility is exemplified by a recent study showing that substance P, a pro-inflammatory mediator with well-established roles in nociception [14], activates brain microvascular endothelial cells. This leads to secretion of tumor necrosis factor alpha (TNFα) and angiopoietin-2 thus changing the localization and distribution of TJ protein zonula occludins-1 and claudin-5 structures as well as increasing permeability of brain microvascular endothelial cells [15]. Moreover, it is well-established that glia play a pivotal role in BBB maintenance [16]. Glial activation and the production of pro-inflammatory cytokines including interleukin-1 beta and TNFα has been demonstrated in many CP models, and these mediators may enhance BBB permeability [17] (Fig. 1).

Besides local immune response in the form of glial activation that might parallel CP in various brain regions [18], the pathological consequences of BBB compromise may result from the disruption of relative “immune quiescence.” The brain is normally accessible only to small, lipid-soluble molecules [19], but in pain states, additional circulating mediators can access it. For instance, in models of surgical trauma, hippocampal BBB disruption and increased levels of systemic cytokines accompany significant neurocognitive impairment [20, 21], potentially through an interleukin-1-beta-dependent mechanism [22]. Circulating autoantibodies could also be involved since they are elevated in various pain conditions including complex regional pain syndrome [23] and back pain [24]. While there is little specific evidence of pain-related brain autoantibody infiltration, this may be a reasonable hypothesis based on observations of such infiltration in conditions such as systemic lupus erythematosus [25] and stroke [26].

(b) Circulating mediators leaking through the BBB create cytogenic and neovascular niches associated with pain-related co-morbidities.

Cytogenic changes may occur in CP in the form of hippocampal neurogenesis in neonates [27] and adults [28]; furthermore CP is associated with reversible anatomical and epigenetic changes in the prefrontal cortex in humans [2] and mice [4]. Based on previous publications [7–13], we hypothesize that this is partially due to BBB compromise throughout some of the brain vasculature, thereby resulting in the aberrant distribution of mediators that are responsible for cytogenesis.

**Fig. 2:**
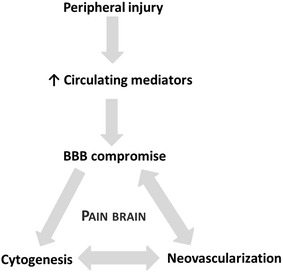
Vicious cycle of BBB compromise and cytogenesis/neovascularization contributes to pain-associated neuroplasticity. *BBB* blood–brain barrier

The bidirectional regulation of neuronal and vascular growth and barrier formation has been investigated in greatest detail during development [29]. Less is known about these processes in the adult brain where plasticity is observed, though our knowledge base is expanding rapidly. For example, local vasculature with a leaky BBB can regulate adult neurogenesis [30] and experience-dependent angiogenesis and changes in neurovascular structure occur in the somatosensory cortex [31] (though pain as an experience was not carefully evaluated). Neurovascular changes were also found to regulate learning and memory in the Morris water maze [32]. These observations are significant as angiogenesis is a localized process during which BBB integrity may be compromised due to the existence of an incomplete epithelium [33] (Fig. 2). We hypothesize that, similar to the observations related to CNS formation, memory formation and experience-related neuroplasticity, CP alters regional vascular structure [34] and BBB permeability. This provides a pathway for the direct communication between the systemic circulation and the brain.

## Testing the hypothesis

Our hypothesis can be tested in both preclinical and clinical settings. In animal models of pain, the existence of BBB compromise, (neo)vascularization and neurogenesis (and their localization relative to one another) can be accomplished by using Evan’s blue/TJ protein quantification/IgG and IgM quantification, India ink-gelatin perfusion, and BrdU staining, respectively. Additionally, undertaking longitudinal studies would be crucial in distinguishing between transient changes after injury and the chronic ones that parallel the timecourse of development of comorbidities in addition to teasing apart cellular proliferation from cellular survival. The advent of sophisticated next-generation microscopy tools [35] could potentially help visualize the connection of astrocytic podocytes to blood vessels in in vitro preparations, and could even be employed to test the effect of different mediators (including those elevated in CP) in the astrocyte-blood vessel preparations. Besides studying the physical integrity of the BBB, its biochemical barrier functionality can be studied in vitro, including the examination of metabolizing enzymes and ATP-driven efflux pumps [36]. Furthermore, the BBB can be experimentally damaged in different brain structures and its long-term effects studied on pain-associated comorbidities following the induction of CP. Such studies could tease apart the effects of BBB compromise and those of circulating mediators in the intact BBB. For instance, pre-existing circulating autoantibodies were shown to exert either beneficial or detrimental effects in ischemic brain injury, depending on the integrity of the BBB [26].

The postmortem study of CP brains could yield equally important information: In suicide completers, for example, a dysfunction in astrocyte connexins is observed in the dorsolateral prefrontal cortex [37], potentially due to a weakened BBB [38]. Similarly, in vivo imaging of BBB disruption in CP patients would be crucial [39], particularly if the association between BBB compromise and cerebral blood flow, pain score, and observed comorbidities is examined in longitudinal timecourse studies.

## Implications of the hypothesis

Understanding changes in BBB permeability in CP conditions has clear implications both in the imaging (delivery of contrast agents) and treatment (delivery of therapeutic agents) of painful conditions. For instance, changes observed by brain imaging could be confounded by BBB compromise and anti-angiogenic agents could be considered potential therapeutic targets in instances where regional leak of the BBB due to neurovascular changes is observed.

It should be recognized that the current hypothesis does not exclude any alternative paths through which immune cells traffic through the CNS that might also modulate chronic pain. For instance, a recent publication showed the existence of a functional CNS lymphatic system, further challenging the long-held assumptions in CNS neuro-immunology [40].

## Abbreviations

BBB: blood–brain-barrier
CNS: central nervous system
CP: chronic pain
TJ: tight junction
TNFα: tumor necrosis factor alpha
